# Compact silicon 10-mode multi/demultiplexer for hybrid mode- and polarisation-division multiplexing system

**DOI:** 10.1038/s41598-019-49763-0

**Published:** 2019-09-13

**Authors:** Weifeng Jiang, Jinye Miao, Tao Li

**Affiliations:** College of Electronic and Optical Engineering, Nanjing University of Posts and Telecommunications, Nanjing, 210023 China

**Keywords:** Integrated optics, Fibre optics and optical communications

## Abstract

To further increase the capacity of the optical transmission system, the hybrid mode- and polarisation-division multiplexing (MDM-PDM) technology has been proved to be an efficient approach by multiplexing dual polarisations for each orthogonal eigen mode. A hybrid (de)multiplexer [(De)MUX] is one of the most important fabrics for the hybrid MDM-PDM networks. A compact silicon 10-mode hybrid (De)MUX is proposed based on three cascaded asymmetric directional couplers (ADCs) based sections, three adiabatic tapers, and a polarisation beam splitter (PBS). The phase-matching conditions can be achieved by varying the widths of the bus waveguides for the TM modes and then by varying the widths of the access waveguides for the TE modes. The simulated results show that a compact total coupling length for TM_1_ ~ TM_3_ and TE_1_ ~ TE_5_ modes can be achieved to be 55.4 μm. In addition, the total loss of the proposed hybrid (De)MUX can be reduced benefitting from the fewer tapers compared with the conventional cascaded ADCs. The PBS is also optimised with a compact length of 7.0 μm and high extinction ratios of 32.9 dB and 15.4 dB for the TM_0_ and TE_0_ modes, respectively.

## Introduction

In the past three decades, the transmission capacity and bandwidth of the optical communication system have been dramatically increased to accommodate exponential network-traffic growth, benefitting from the dense wavelength-division multiplexing (DWDM) technology^1^. However, the capacity of the traditional single-mode fibre based DWDM system is restricted by the Shannon limits of nonlinear fibre transmission. In order to further improve the capacity, two multiplexing technologies, mode- and polarisation-division multiplexing (MDM and PDM) have been emerging as promising approaches to overcome the capacity crunch, which can achieve the possible capacity of Peta-bit/s^2,3^. Deploying the MDM by using *M* orthogonal eigen modes for each DWDM wavelength carries *M* times the capacity. In addition, multiplexing dual polarisations for each orthogonal eigen mode achieves 2 × *M* times the capacity for hybrid WDM, MDM, and PDM systems. In order to build this hybrid multiplexing system, hybrid multi/demultiplexers (MUX/DeMUXs) are of great importance.

Recently, silicon photonics based polarisation and mode (De)MUXs have been extensively reported for on-chip PDM and MDM sub-systems, respectively, which can yield ultra-compact, CMOS-compatible, and low-cost components^4,5^. Two approaches, mode evolution and mode coupling have been proposed to realise both the polarisation and mode (De)MUXs^6,7^. In view of the mode evolution approach, many adiabatic couplers (ACs)^8,9^, multimode interference (MMI) couplers^10,11^, topology structures^12,13^, and Y-branches^14,15^ based architectures have been reported to build a polarisation (De)MUX or a mode (De)MUX. Although broad bandwidths and high extinction ratios (ERs) can be achieved for these devices, the footprints are relatively large owing to the mode evolution process. For the mode coupling approach, various asymmetric directional couplers (ADCs)^16,17^, grating-assisted ADCs^18,19^, and densely packed waveguide arrays (DPWAs)^20,21^ have been demonstrated to build ultra-compact polarisation and mode (De)MUXs. Among these reported (De)MUXs, however, two polarisations of only one fundamental mode were handled in a polarisation (De)MUX or two modes of only single polarisation were supported in a mode (De)MUX. A compact, scalable, and broadband (De)MUX supporting more operating modes on dual polarisations is essential to realise on-chip hybrid multiplexing systems.

In order to excite more modes, micro-ring resonators^22^, cascaded ADCs^23^, grating-assisted ADCs^24^, and cascaded dual-core ACs^25^ have been reported. Lipson *et al*. experimentally demonstrated a 3-mode (De)MUX, comprising of two tapers, three identical micro-rings, and three ADCs for hybrid MDM and WDM^22^. Dai *et al*. proposed and demonstrated a 4-mode (De)MUX for the TM polarisation based on three cascaded ADCs and three tapers^23^. A 4-mode narrow-band (De)MUX was also optimised for the TE polarisation via grating-assisted ADCs^24^. Dai *et al*. experimentally demonstrated an 8-mode hybrid (De)MUX, consisting of six cascaded-ADCs, six tapers, and a polarisation beam splitter (PBS), which enables simultaneous MDM and PDM^26,27^. More recently, Dai *et al*. employed five cascaded dual-core ACs and five PBSes to extend their hybrid (De)MUX to handle 10-mode for both the TE and TM polarisations^25^, TM_0_ ~ TM_3_ and TE_0_ ~ TE_5_. Su *et al*. proposed and experimentally demonstrated a 11-mode (De)MUX for the TE polarisation based on ten cascaded-ADCs with subwavelength gratings (SWGs) and ten tapers^28^. We can see that the cascaded-ADC based architecture is the most popular approach for realising a hybrid (De)MUX, benefiting from its simple structure, easy scalability, and elasticity to (de)multiplex any desired high-order mode. However, for traditional hybrid-(De)MUXs based on the cascaded ADCs, each ADC is used to handle only one mode and an essential taper must be added between two adjacent cascaded-ADCs. Sequentially, the size of the total device is not compact and the excess loss would be induced due to the additional tapers. A more compact (De)MUX with fewer tapers is desired for both the on-chip hybrid multiplexing systems and optical fibre transmission systems.

In this paper, we propose and optimise a 10-mode hybrid (De)MUX for both the TE and TM polarisations, TM_0_ ~ TM_3_ and TE_0_ ~ TE_5_, based on three cascaded ADCs based sections, three adiabatic tapers, and a PBS, which can enable hybrid MDM-PDM. Different from the conventional cascaded-ADCs, each ADC-section can simultaneously handle two or three modes based on the multi-phase matching conditions. On the contrary, if this proposed 10-mode hybrid-(De)MUX is built by using the conventional cascaded-ADCs, eight ADCs and eight tapers must be implemented, which would induce both the larger footprint and additional loss. Therefore, the number of the required tapers can be dramatically cut down based on the proposed schematic. In this case, the modal and propagation characteristics are studied by using the full-vectorial finite element method (FV-FEM) and 3D full-vectorial finite difference time domain (3D-FV-FDTD) method, respectively.

## Results

### Schematic and Principle

**Figure 1:**
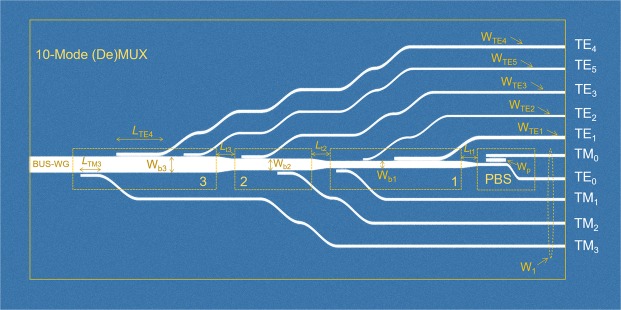
Schematic/layout of the proposed 10-mode hybrid (de)multiplexer for both the TE and TM polarisations. Three ADCs based section 1 is capable of handling the TM_1_, TE_1_, and TE_2_ modes. Two ADCs based section 2 is handling the TM_2_ and TE_3_ modes. Three ADCs based section 3 is handling the TM_3_, TE_4_, and TE_5_ modes. A polarisation beam splitter (PBS) section is for splitting the TM_0_ and TE_0_ modes. Only 3 tapers are implemented in between these four sections based on the improved phase-matching condition for asymmetric directional couplers.

**Figure 2:**
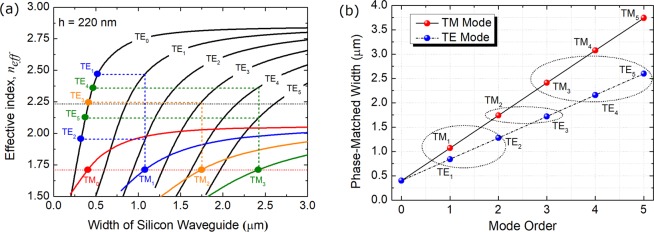
(**a**) Variations of the effective index with the width of the silicon waveguide; (**b**) Phase-matched widths of bus waveguides for both the TE and TM polarisations.

The schematic diagram of the proposed 10-mode hybrid (De)MUX with dual polarisations is shown in Fig. 1, consisting of three cascaded ADCs based sections, three tapers, and a PBS. The widths of bus waveguides (WGs) are denoted by W_bi_ for *i*th ADC-section, where *i* = 1, 2, and 3. The lengths of three tapers are denoted by *L*_ti_ for *i*th taper. The coupling lengths of the TM and TE polarisations are represented by *L*_TMi_ and *L*_TEj_, respectively, where j = 1, 2, 3, 4, 5. The widths of the access WGs for the TM_0_ ~ TM_3_ and TE_0_ modes are identical and denoted by W_1_, while those for the TE_1_ ~ TE_5_ modes are denoted by W_TEj_. It can also be observed from Fig. 1 that a triple-WG coupler (TWC) based PBS is implemented with the central-WG width of W_p_. This PBS is used to separate or combine the TM_0_ and TE_0_ modes. For three cascaded ADCs based sections, the bus-WG widths are determined according to the phase-matching conditions for the TM_1_, TM_2_, and TM_3_ modes, respectively. Sequentially, the phase-matched widths of the access WGs for the TE_1_ ~ TE_5_ modes are determined according to the phase-matched bus-WG widths for three TM modes, which will be explained in Fig. 2. The first ADC-section is capable of handling the TM_1_, TE_1_, and TE_2_ modes. The second ADC-section is for handling the TM_2_ and TE_3_ modes. The third ADC-section is for handling the TM_3_, TE_4_, and TE_5_ modes.

The phase-matching conditions for three cascaded ADCs based sections are studied and shown in Fig. 2. Variations of the effective index with the width of the silicon waveguide are calculated by using the FV-FEM and shown in Fig. 2(a). In this case, the thickness of all silicon WGs with the silica cladding is set as h = 220 nm. The refractive indices of the silicon and silicon oxide are set to be 3.47548 and 1.46, respectively at the wavelength of 1550 nm. The access width, W_1_, is chosen to be 400 nm for single-mode operation. The phase-matched bus-WG widths (W_b1_, W_b2_, and W_b3_) of 1^st^, 2^nd^, and 3^rd^ ADC-sections are determined by the phase-matching conditions for the TM_1_, TM_2_, and TM_3_ modes, respectively, denoted by the horizontally blue, orange, and green dots in Fig. 2(a). It can be noted from Fig. 2(a) that the bus WG of the first ADC-section can support the TE_0_ ~ TE_2_ and TM_0_ ~ TM_1_ modes. That of the second ADC-section can support the TE_0_ ~ TE_4_ and TM_0_ ~ TM_2_ modes. That of the third ADC-section can support the TE_0_ ~ TE_5_ and TM_0_ ~ TM_3_ modes. The phase-matched access-WG widths of both the TE_1_ and TE_2_ modes, W_TE1_ and W_TE2_, are obtained according to the phase-matched W_b1_ of the first ADC-section, which are denoted by two blue dots in Fig. 2(a). The phase-matched W_TE3_ is determined by the phase-matched W_b2_ of the second ADC-section, denoted by an orange dot. The phase-matched W_TE4_ and W_TE5_ are determined by the phase-matched W_b3_ of the third ADC-section, denoted by two green dots. Although the phase-matched access-WG widths of TE_1_ ~ TE_3_ modes can also be obtained with the phase-matched W_b3_ of the third ADC-section, these matched widths cannot assure the single-mode operation for these access WGs. To further explain this choice, we plot the phase-matched widths of bus WGs for the TE_1_ ~ TE_5_ and TM_1_ ~ TM_5_ modes in Fig. 2(b) for an identical silicon WG with the size of W_1_ × h = 400 × 220 nm^2^. It can be noted that if the phase-matched bus-WG widths are close to each other, these specific modes can be handled by using a same ADC-section. In addition, the bus-WG widths are determined by the phase-matched TM modes, while those can also be determined by the phase-matched TE modes. This principle can not only be applied for 10 modes in this case, but also can be extended to any higher-order mode for hybrid MDM-PDM systems.

### Phase-Matching Conditions for TM Modes

**Figure 3:**
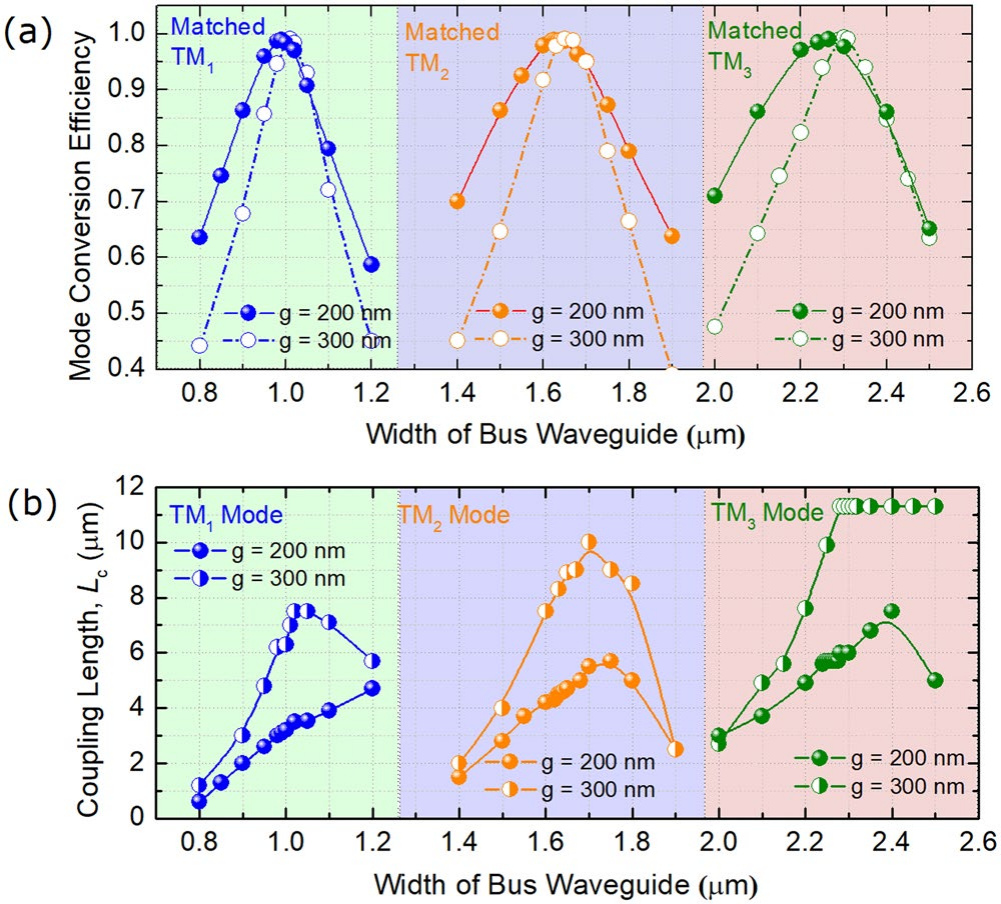
Phase-matching conditions for TM_1_, TM_2_ and TM_3_ modes. Variations of (**a**) the mode conversion efficiency and (**b**) coupling length with the width of the bus waveguide.

**Figure 4:**
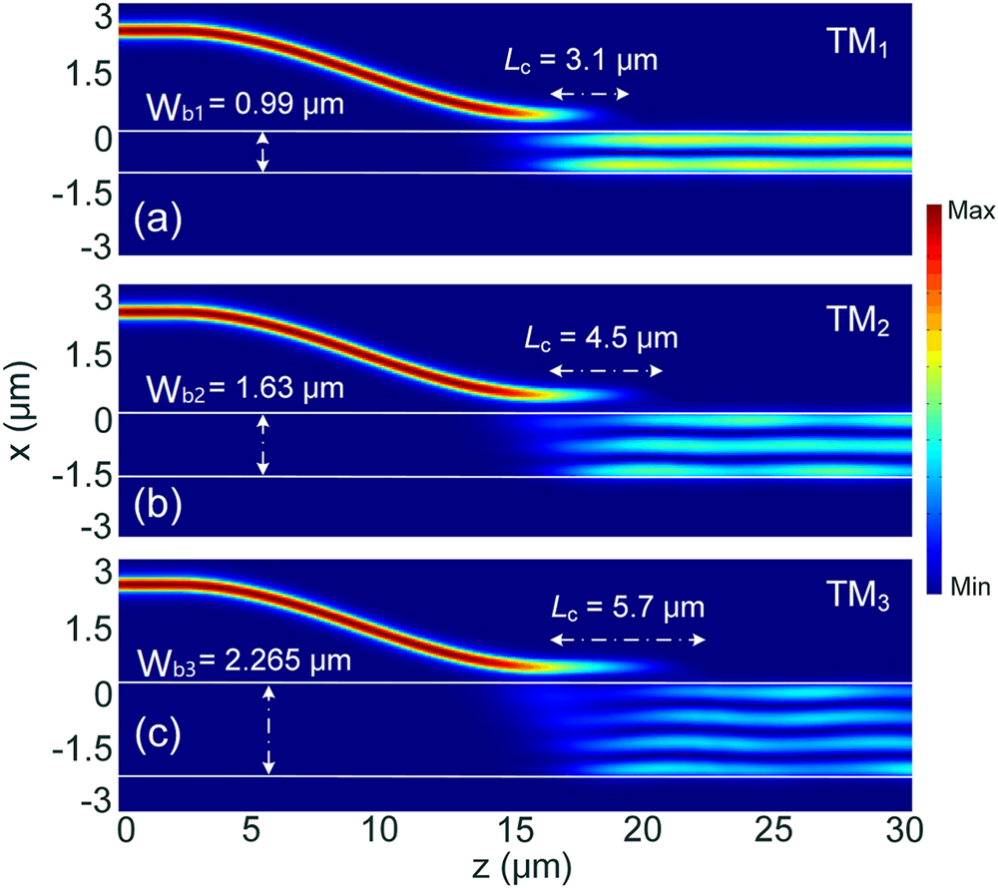
Propagation fields of the Poynting vector for matched (**a**) TM_1_, (**b**) TM_2_, and (**c**) TM_3_ modes, respectively.

Although phase-matching conditions for three TM modes have been obtained from Fig. 2 for isolated WGs, those for the composite WGs of ADCs should be studied, which may shift dramatically due to the strong coupling effect. In this Section, phase-matching conditions for three TM modes are calculated by using the 3D-FV-FDTD method, as shown in Fig. 3. As the width of the access WG is set as W_1_ = 400 nm for each TM mode, the widths of the bus WGs are varied to achieve the best mode conversion efficiency (MCE). In the calculations, the gaps between the access and bus WGs are varied for g = 200 and 300 nm. It can be noted from Fig. 3(a) that the MCEs for both the gaps can achieve to be >98.8% at the phase-matching points for three TM modes. The MCEs are decreased from the phase-matching points to both the wider and narrower bus-WG widths. Variations of the coupling lengths, *L*_c_ with the width of the bus WG are shown in Fig. 3(b) for both g = 200 and 300 nm. It can be noted that a shorter coupling length can be obtained with a narrower gap for three TM modes. In this case, the gap between the access and bus WGs is set as g = 200 nm, which can provide both the high MCEs of 0.989, 0.988, 0.99 and compact coupling-lengths of 3.1, 4.5, and 5.7 μm for TM_1_, TM_2_, and TM_3_ modes, respectively. The widths of the bus WGs are set to be W_b1_ = 0.99 μm, W_b2_ = 1.63 μm, and W_b3_ = 2.265 μm for the first, second, and third ADC-sections according to the phase-matching conditions for the TM_1_, TM_2_, and TM_3_ modes, respectively. The propagation fields of the Poynting vector under the phase-matching conditions for three TM modes are calculated by using the 3D-FV-FDTD method and shown in Fig. 4. It can be observed that the input fundamental TM mode from the access WGs can be completely converted to the matched high-order modes in the bus WGs and vice versa.

### Phase-Matching Conditions for TE Modes

**Figure 5:**
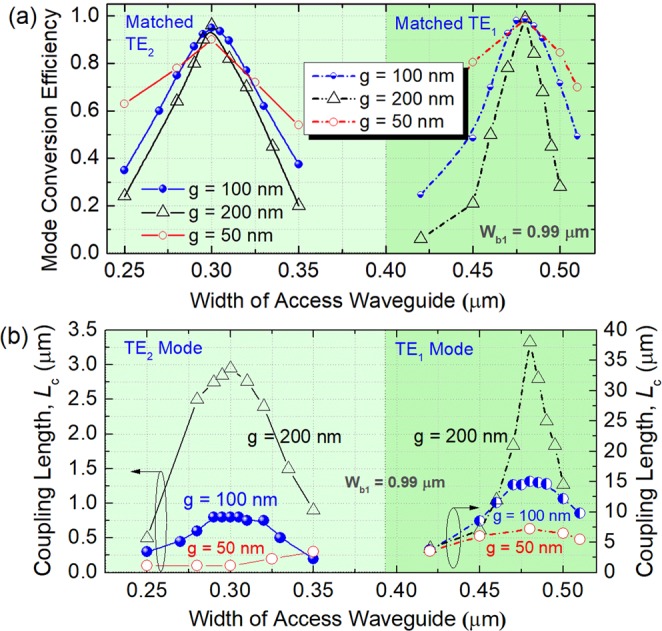
Phase-matching conditions for TE_1_ and TE_2_ modes. Variations of (**a**) mode conversion efficiency and (**b**) coupling length with the width of the access waveguide.

As the widths of the bus WGs have been obtained in the above section, the widths of the access WGs of three ADC-sections are varied to achieve the phase-matching conditions for the TE_1_ ~ TE_5_ modes. As stated above, the first ADC-section is for (de)multiplexing the TM_1_, TE_1_, and TE_2_ modes. Variations of the MCEs with the width of the access WG are shown in Fig. 5(a) for both the TE_1_ and TE_2_ modes. The MCEs of the TE_1_ and TE_2_ modes can achieve to be 0.988 and 0.95 for the phase-matched access-WG widths of W_TE1_ = 0.48 μm and W_TE2_ = 0.3 μm, respectively for g = 100 nm. Variations of the coupling lengths with the widths of the access WGs are shown in Fig. 5(b) for the gap, g = 100 nm. It can be noted that the coupling lengths of the TE_1_ and TE_2_ modes are 15.0 and 0.8 μm, respectively under the phase-matching conditions.

**Figure 6:**
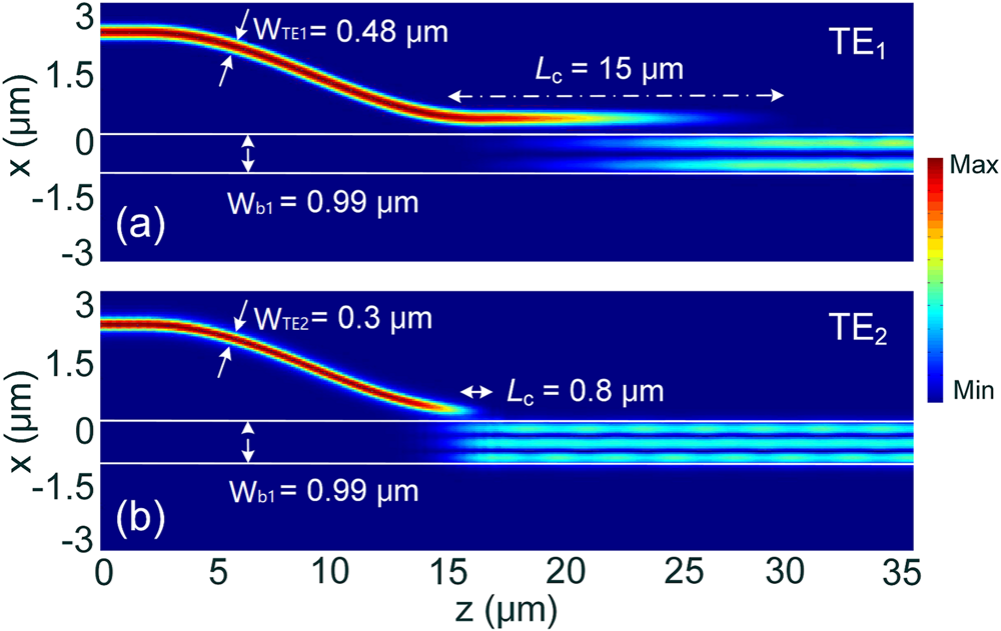
Propagation fields of the Poynting vector for matched (**a**) TE_1_ and (**b**) TE_2_ modes, respectively.

Generally, a narrower gap would lead to a compact coupling-length due to a stronger coupling-strength between two waveguides, but it also induces a larger crosstalk (CT) and a lower MCE. On the contrary, a lower CT and a higher MCE can be obtained with a wider gap, but this would increase the coupling length. We also calculate the variations of the MCE and coupling length for g = 50 and 200 nm, as shown in Fig. 5. It can be noted that by increasing the gap from g = 100 nm to 200 nm, the MCE of the TE_1_ mode is slightly increased from 0.988 to 0.99, but the coupling length is dramatically increased from 15 μm to 38 μm. For the TE_2_ mode, the MCE is enhanced to 0.96, but the coupling length is increased to 2.95 μm. For the gap, g = 50 nm, the coupling lengths of the TE_1_ and TE_2_ modes are significantly reduced to 7.2 and 0.1 μm with a slight MCE-decrease <0.05. However, the fabrication of a narrow gap, g = 50 nm is relatively difficult. In this case, the gap, g = 100 nm is chosen for both the TE_1_ and TE_2_ modes, thereby both the compact coupling-lengths and high MCEs can be achieved without a demanding fabrication-process. The propagation fields of the Poynting vector under the phase-matching conditions are calculated for the TE_1_ and TE_2_ mode, as shown in Fig. 6(a,b), respectively. It can be observed that the input fundamental TE modes are totally transferred from the access WGs to the TE_1_ and TE_2_ modes of the bus WG in the first ADC-section.

**Figure 7:**
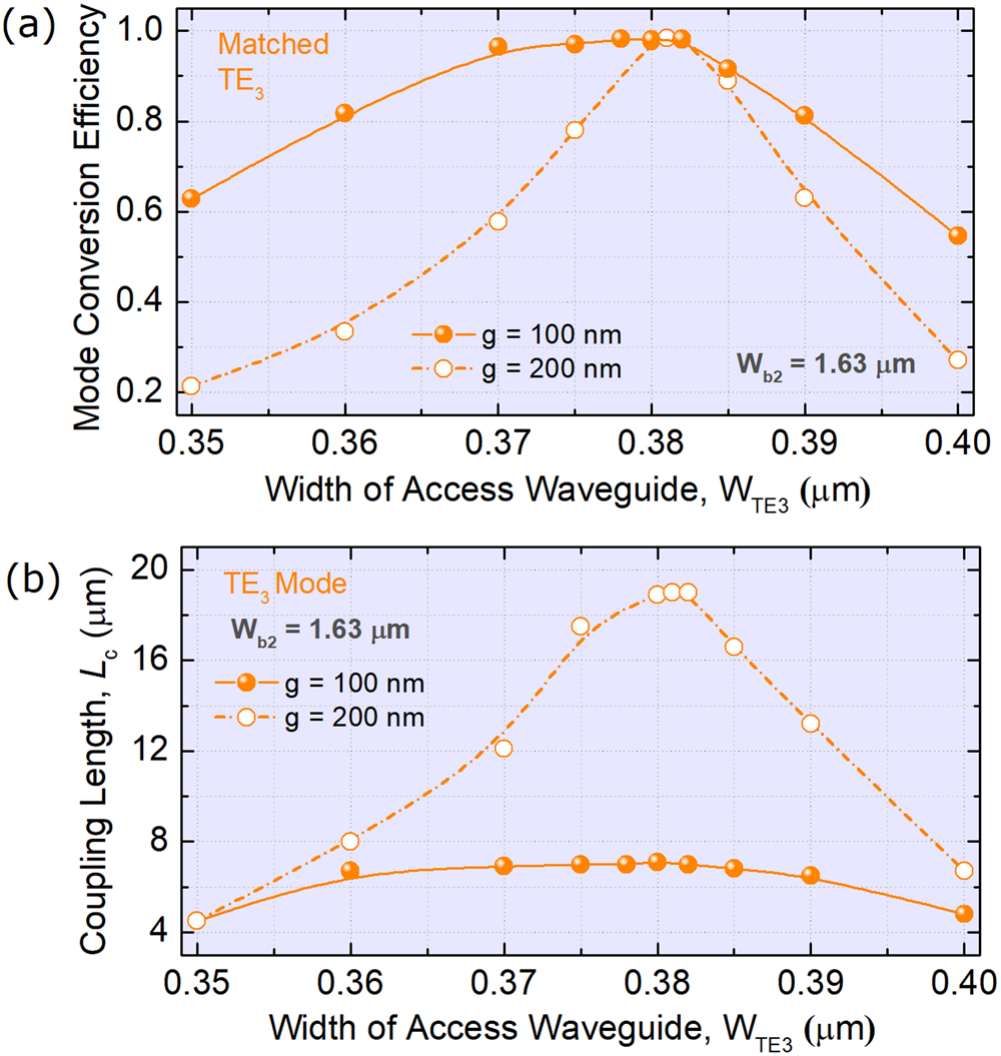
Phase-matching conditions for the TE_3_ mode. Variations of (**a**) the mode conversion efficiency and (**b**) coupling length with the width of the access waveguide.

**Figure 8:**
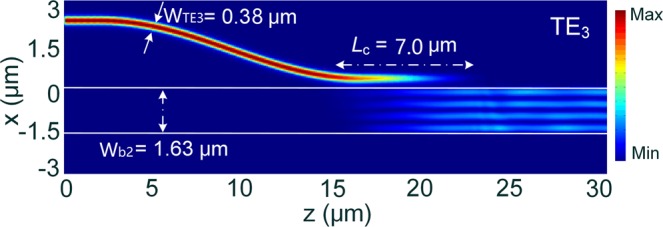
Propagation field of the Poynting vector for the matched TE_3_ mode.

Next, the phase-matching condition for the TE_3_ mode in the second ADC-section is studied by using the 3D-FV-FDTD method. Variations of the MCEs and coupling lengths of the TE_3_ mode with the width of the access WG, W_TE3_ are shown in Fig. 7(a,b), respectively. It can be noted from Fig. 7(a) that the MCEs are 0.981 and 0.984 for the gap, g = 100 and 200 nm, respectively under the phase-matching conditions. It can be noted from Fig. 7(b) that the coupling lengths are calculated to be *L*_c_ = 7.0 and 19.0 μm for g = 100 and 200 nm, respectively. In addition, a narrower gap can not only provide a compact coupling-length, but also achieve a flat curve. In this case, the gaps between the access and bus WGs of three ADC-sections are chosen to be g = 100 nm for the TE_1_ ~ TE_5_ modes. The propagation field of the Poynting vector of the TE_3_ mode along z axis is shown in Fig. 8, which shows the high performance of the mode conversion between the access and bus WGs in the second ADC-section under the phase-matching condition.

**Figure 9:**
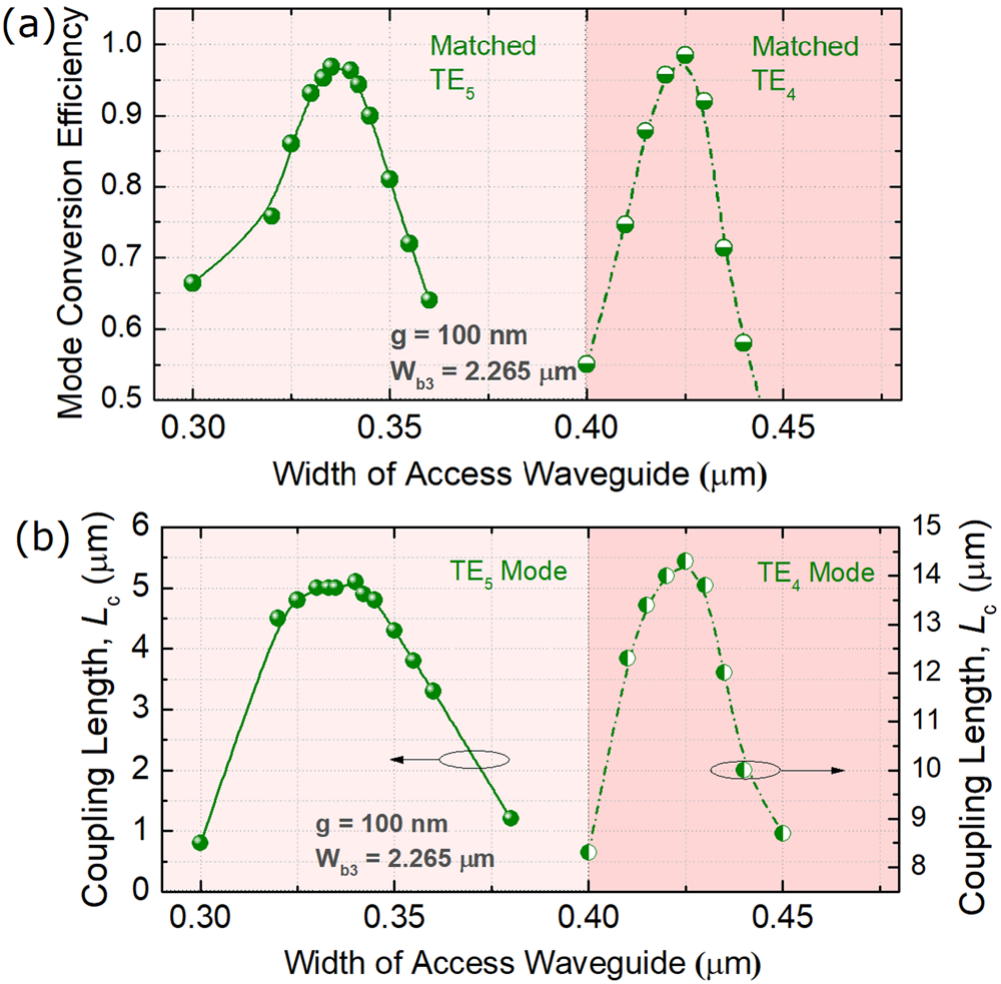
Phase-matching conditions for TE_4_ and TE_5_ modes. Variations of (**a**) the mode conversion efficiency and (**b**) coupling length with the width of the access waveguide.

**Figure 10:**
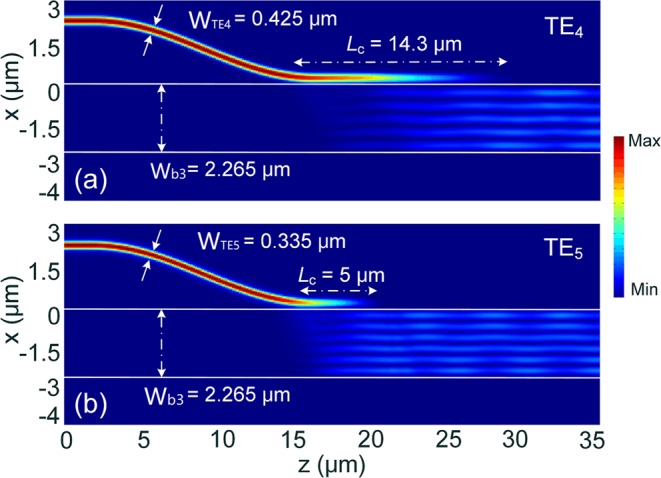
Propagation fields of the Poynting vector for matched (**a**) TE_4_ and (**b**) TE_5_ modes, respectively.

Next, the phase-matching conditions for the TE_4_ and TE_5_ modes in the third ADC-section are studied by using the 3D-FV-FDTD method. Variations of the MCEs and coupling lengths of the TE_4_ and TE_5_ modes with the widths of the access WGs are shown in Fig. 9(a,b), respectively. Under the phase-matching conditions of W_TE4_ = 0.425 μm and W_TE5_ = 0.335 μm, the MCEs and coupling lengths are calculated to be MCE = 0.984 and 0.968, *L*_c_ = 14.3 and 5.0 μm, respectively. The propagation fields along z axis are shown in Fig. 10(a,b) for the TE_4_ and TE_5_ modes, respectively. It can be observed that the mode coupling and conversion processes for both the TE modes can be achieved from the fundamental TE mode to the desired high-order modes for the gap, g = 100 nm.

### Operation, bandwidth, and fabrication-tolerance for cascaded ADC-sections

The parameters and performance of three cascaded ADCs based sections have been optimised and summarised in Table 1. It can be noted that the first, second, and third ADC-sections can be used to (de)multiplex the TM_1_, TE_1_, and TE_2_ modes; TM_2_ and TE_3_ modes; TM_3_, TE_4_, and TE_5_ modes, respectively. The MCE is above 0.95 for all modes, which can achieve a high performance for the proposed 10-mode hybrid (De)MUX. The total coupling length for three cascaded ADC-sections is calculated to be 55.4 μm, which is an ultra-compact size compared to the length of >300 μm for the reported 10-mode (De)MUX based on the cascaded dual-core ACs^25^. Furthermore, our proposed 10-mode hybrid (De)MUX is also more compact than reported 8-mode hybrid (De)MUX and 11-mode (De)MUX based on conventional cascaded-ADCs^26–28^.

**Table 1:** Phase-matched parameters and performance for the cascaded ADC-sections.

ADC-Section	1			2		3		
Modes	TM_1_	TE_1_	TE_2_	TM_2_	TE_3_	TM_3_	TE_4_	TE_5_
W_bus_ (μm)	0.99			1.63		2.265		
Gap, g (nm)	200	100	100	200	100	200	100	100
W_access_ (μm)	0.4	0.48	0.3	0.4	0.38	0.4	0.425	0.335
*L*_c_ (μm)	3.1	15	0.8	4.5	7.0	5.7	14.3	5.0
MCE (%)	98.9	98.8	95.0	98.8	98.1	99.0	98.4	96.8

**Figure 11:**
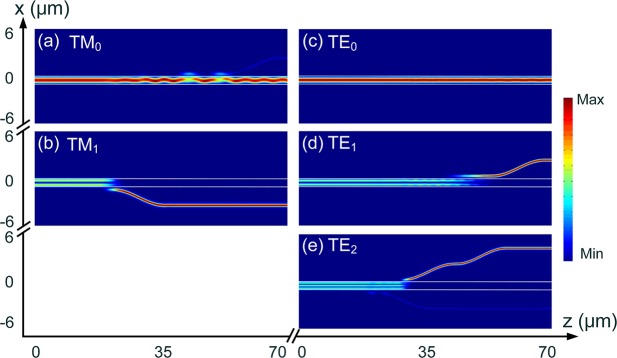
Propagation fields of the Poynting vector for first ADC-section (section 1 in Fig. 1), (de)multiplexing the TM_1_, TE_1_, and TE_2_ modes.

Next, the performance of three cascaded ADCs based sections are studied by using the 3D-FV-FDTD method. The combined structure of three ADC-sections is shown in Fig. 1. For the first ADC-section (section 1 in Fig. 1), the TM_1_, TE_2_ and TE_1_ modes are sequentially demultiplexed. The propagation fields of the Poynting vector for the first ADC-section are shown in Fig. 11. As the first ADC-section can support the TM_0_ and TE_0_ modes, we also calculated the propagation fields of these two fundamental modes. It can be observed from Fig. 11 that the input TM_0_ mode propagates through the TM_1_ and TE_2_ coupling-sections, but slightly couples to the TE_1_ access-WG and then couples back to the bus WG. Although a slight coupling-power of the TM_0_ mode to the TE_1_ access-WG, a low CT of <−15 dB can be achieved. It can also be observed from Fig. 11 that the TM_1_, TE_1_, and TE_2_ modes are completely demultiplexed from the bus-WG. When launching the TE_0_ mode into the bus-WG, it propagates along the bus-WG without any mode-coupling to any access-WG.

**Figure 12:**
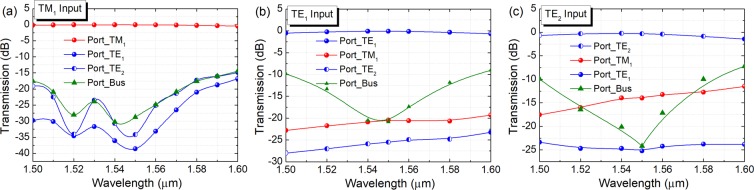
Operation bandwidth of first ADC-section. Variations of the transmittance with the wavelength for inputting (**a**) TM_1_, (**b**) TE_1_, and (**c**) TE_2_ modes, respectively.

The operation bandwidths of the first ADC-section are studied and shown in Fig. 12(a–c), for inputting the operating TM_1_, TE_1_, and TE_2_ modes, respectively. It can be noted from Fig. 12(a) that when launching the TM_1_ mode into the bus WG, the insertion loss (IL) of the TM_1_ mode is lower than 0.46 dB for a broad bandwidth of 100 nm from 1500 nm to 1600 nm. The mode CTs for other channels of the first ADC-section are lower than −15 dB over a 100 nm bandwidth. It can be noted from Fig. 12(b) that when launching the TE_1_ mode, the IL of the TE_1_ mode is less than 0.66 dB. The mode CT of the access WG of the TM_1_ mode is lower than −19.3 dB and that of the TE_2_ mode is lower than −23.2 dB over a 100 nm bandwidth. The mode CT of the bus WG is <−15 dB for the operation wavelength from 1523 nm to 1569 nm. As shown in Fig. 12(c), the IL of the TE_2_ mode is <1.0 dB with the wavelength in between 1500 nm and 1588 nm, when launching the TE_2_ mode into the bus-WG. The mode CT of the TM_1_ mode is lower than −11.5 dB over the 100 nm bandwidth and that of the TE_1_ mode is <−23.3 dB. The mode CT of the bus-WG is lower than −10 dB for the wavelength from 1500 nm to 1580 nm. The worst CT of −10 dB is mainly due to the residual power in the bus waveguide. However, the residual power of any high-order mode in the bus waveguide can be eliminated via three adiabatic tapers and only the fundamental modes can propagate through the PBS. Hence, the CT of our proposed hybrid (De)MUX should be discussed between the access-waveguides. As shown in Fig. 12, when operating over the C-band, the mode CTs are lower than −22.7 dB, −20.5 dB, and −13.1 dB for inputting the TM_1_, TE_1_, and TE_2_ modes, respectively. As the mode CT for inputting TE_2_ mode may be larger than −15 dB in the C-band, this mode CT can be further reduced by cascading a TM-pass polariser at the output port of the access waveguide for the TM_1_ mode. This polariser can eliminate the unwanted TE-polarised light power. Such TM-pass polariser^29^ has been demonstrated with a low IL ≤0.5 dB and a low CT ≤−27 dB. By using this configuration, the mode CT for inputting TE_2_ mode can be dramatically reduced.

**Figure 13:**
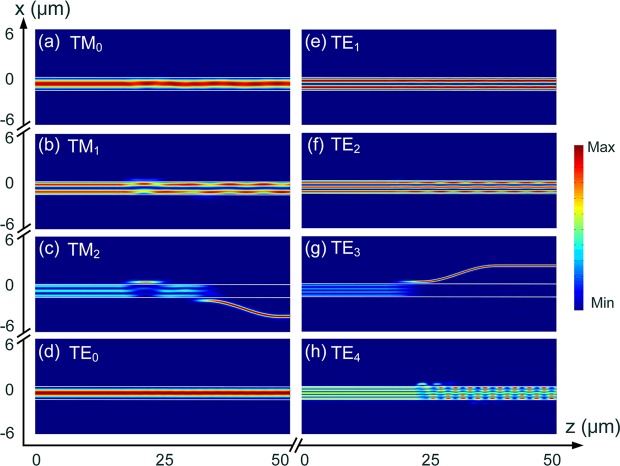
Propagation fields of the Poynting vector for second ADC-section, (de)multiplexing the TM_2_ and TE_3_ modes.

**Figure 14:**
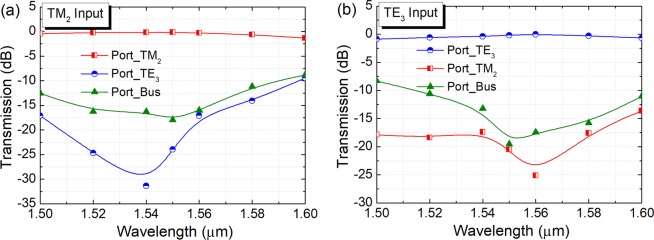
Operation bandwidth of second ADC-section. Variations of the transmittance with the wavelength for inputting (**a**) TM_2_ and (**b**) TE_3_ modes, respectively.

Next, the propagation performance of the second ADC-section for (de)multiplexing the TM_2_ and TE_3_ modes are studied by using the 3D-FV-FDTD method. The propagation fields of the Poynting vector for the second ADC-section are shown in Fig. 13 for the supporting TM_0_ ~ TM_2_ and TE_0_ ~ TE_4_ modes. It can be observed that the input TM_0_, TM_1_, TE_0_ ~ TE_2_ and TE_4_ modes propagate through the bus-WG without mode-coupling, while the TM_2_ and TE_3_ modes are demultiplexed from the bus-WG to the access WGs. It should be noted that as shown in Fig. 13(c), the input TM_2_ mode is firstly coupled to the access-WG and then totally coupled back without any power loss. The operation bandwidths of the second ADC-section are calculated and shown in Fig. 14. It can be noted from Fig. 14(a) that when launching the TM_2_ mode into the bus WG of the second ADC-section, the IL of the TM_2_ mode is less than 1.0 dB from 1500 nm to 1592 nm. The mode CT of the TE_3_ mode is lower than −10 dB over the 100 nm bandwidth, while that of the bus-WG is <−9.0 dB. It can also be noted from Fig. 14 that the IL of the TE_3_ mode is <0.88 dB when inputting the TE_3_ mode into the bus-WG of the second ADC-section. The mode CT is lower than −13.6 dB for the TM_2_ mode over the 100 nm bandwidth and that of the bus-WG is lower than −10 dB for the wavelength from 1514 nm to 1600 nm. When operating over the C-band, the mode CTs are lower than −16.8 dB and −17.4 dB for inputting the TM_1_ and TE_3_ modes, respectively.

**Figure 15:**
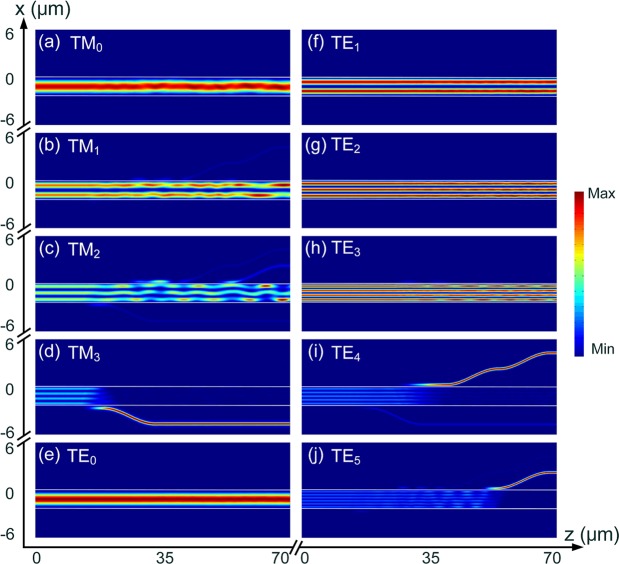
Propagation fields of the Poynting vector for third ADC-section, (de)multiplexing the TM_3_, TE_4_, and TE_5_ modes.

**Figure 16:**
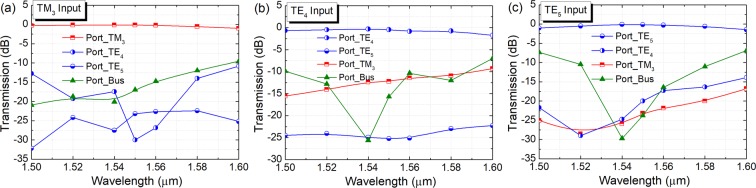
Operation bandwidth of third ADC-section. Variations of the transmittance with the wavelength for inputting (**a**) TM_3_, (**b**) TE_4_, and (**c**) TE_5_ modes, respectively.

Next, the propagation performance of the third ADC-section for (de)multiplexing the TM_3_, TE_4_, and TE_5_ modes are investigated. The propagation fields of the Poynting vector for the third ADC-section are shown in Fig. 15 for TM_0_ ~ TM_3_ and TE_0_ ~ TE_5_ modes. It can be observed that the input TM_0_ ~ TM_2_ and TE_0_ ~ TE_3_ modes propagate through the bus WG of the third ADC-section, while the TM_2_, TE_4_, and TE_5_ modes are completely transferred from the bus WG to the access WGs. It should be noted that when launching the TM_2_ mode, there are slight mode-coupling between the bus WG and three access WGs, which may induce increased mode CTs of these access WGs. To further reduce the mode CTs of these three access-WGs, three polarisers could be implemented for each channel. The operation bandwidths of the third ADC-section are studied and shown in Fig. 16. It can be noted from Fig. 16(a) that when launching the TM_3_ mode into the bus-WG, the IL of the TM_3_ mode is lower than 1.0 dB for the 100 nm bandwidth. The mode CTs of the TE_4_ mode and bus WG are lower than −10 dB and that of the TE_4_ mode is <−22.4 dB over the 100 nm bandwidth. It can also be noted from Fig. 16(b) that the IL of the TE_4_ mode is <1.0 dB with the operation wavelength from 1500 nm to 1585 nm, when inputting the TE_4_ mode. The mode CT of the TE_5_ mode is lower than −22.3 dB over the 100 nm bandwidth and that of the TM_3_ mode is <−10 dB for the wavelength from the 1500 nm to 1594 nm. The mode CT is lower than −10 dB for the bus WG with the operating wavelength from 1500 nm to 1588 nm. As shown in Fig. 16(c), the IL of the TE_5_ mode is <1.0 dB for the wavelength from 1500 nm to 1592 nm. The mode CT of the TE_4_ mode is lower than −14 dB over the 100 nm bandwidth and that of the TM_3_ mode is lower than −16.8 dB. The mode CT of the bus WG is lower than −10 dB for the wavelength from 1516 nm to 1585 nm. When operating over the C-band, the mode CTs are lower than −17.5 dB, −11.4 dB, and −17 dB for inputting the TM_3_, TE_4_, and TE_5_ modes, respectively. A TM-pass polariser^29^ can also be implemented at the output port of the access waveguide for the TM_3_ mode to further reduce the mode CT.

**Figure 17:**
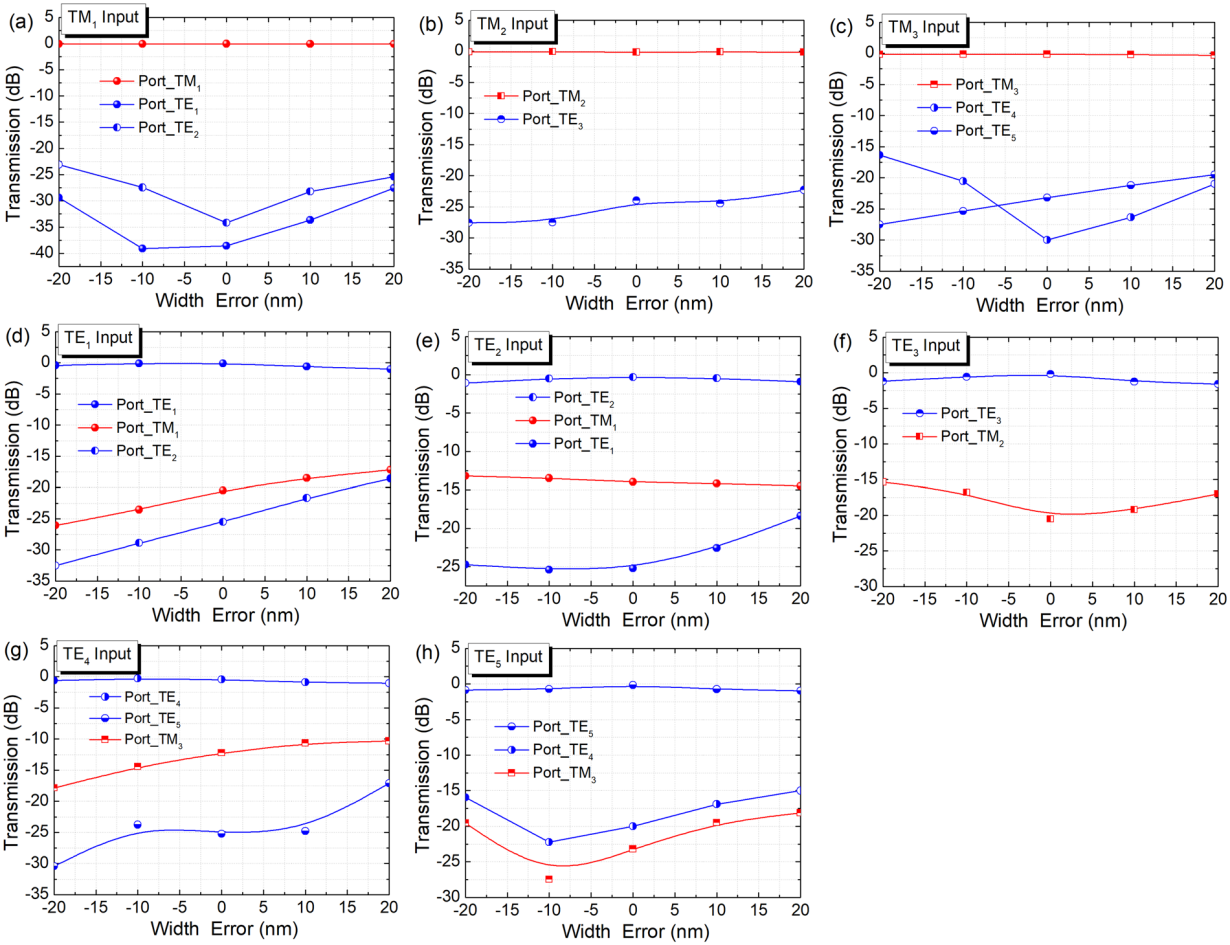
Fabrication tolerance of the cascaded ADC-sections. Variations of the transmittance with the width error for inputting (**a**) TM_1_, (**b**)TM_2_, (**c**) TM_3_, (**d**)TE_1_, (**e**)TE_2_, (**f**)TE_3_, (**g**) TE_4_, and (**h**) TE_5_ modes.

Although the CMOS technology is very mature, some changes can be expected from the optimised designed parameters of our proposed hybrid (De)MUX. A fabrication error of ±20 nm may be induced by the lithography process for most foundries. We calculate the variations of the performance of the optimised hybrid (De)MUX with the waveguide width-error. In the numerical calculations, the width error was applied to the widths of both the bus and access waveguides, but the pitch between these two waveguides was kept constant. Variations of the transmittance with the width error are shown in Fig. 17 for inputting the TM_1_ ~ TM_3_ and TE_1_ ~ TE_5_ modes. It can be noted that with a ±20 nm width-error, the deteriorations of the IL are less than 0.05, 0.02, 0.16, 0.9, 0.78, 1.4, 0.58, and 0.78 dB for inputting the TM_1_ ~ TM_3_ and TE_1_ ~ TE_5_ modes, respectively. We can state that the IL of the proposed hybrid (De)MUX is tolerant to the width error of ±20 nm. In addition, the mode CTs are less than −23, −22.1, −16.2, −16.2, −12.1, −14.2, −10.3, and −14 dB for inputting the TM_1_ ~ TM_3_ and TE_1_ ~ TE_5_ modes, respectively with a ±20 nm width-error. Particularly, the CTs of the TE_2_ ~ TE_5_ modes can be reduced to be <−15 dB by introducing a TM-pass polariser^29^ at the output ports of the access WGs for the TM_1_, TM_2_, and TM_3_ modes, respectively. Another approach could be the use of a narrower gap between the bus and access WGs, thereby the transmittance is more tolerant to the width error compared with a wider gap due to the stronger coupling-strength.

### Optimisation for tapers

**Figure 18:**
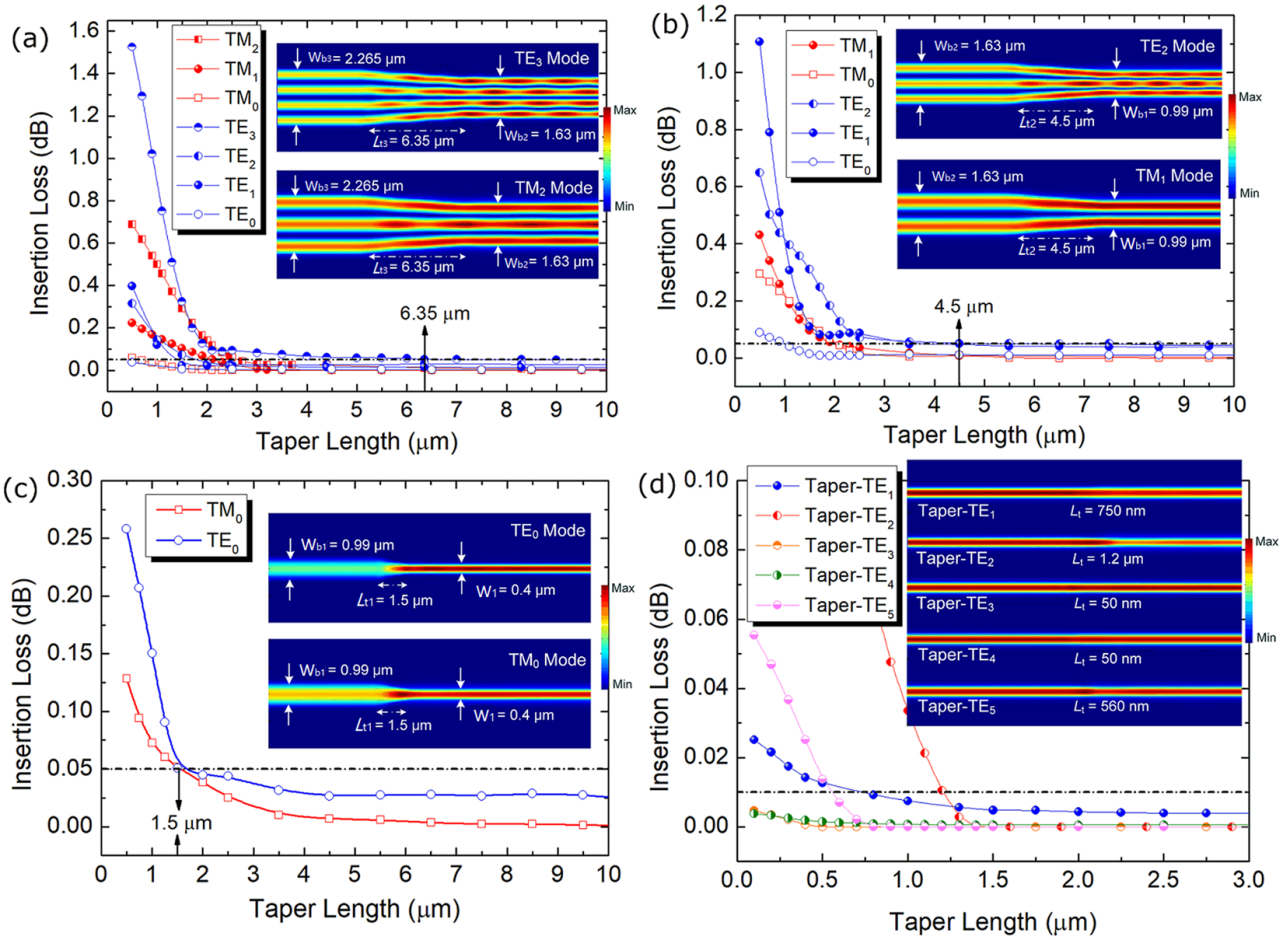
Variations of the insertion loss with the taper lengths of (**a**–**c**) 3 tapers for bus waveguide and (**d**) 5 tapers for access waveguides.

The optimisation for the design of the tapers between the adjacent ADC-sections and PBS are studied by using the 3D-V-FDTD method. Variations of the IL with the taper length of the third taper between the second and third ADC-sections are shown in Fig. 18(a). The ILs of the TM_0_ ~ TM_2_ and TE_0_ ~ TE_3_ modes were calculated due to these modes supported in the bus-WG of the second ADC-section. In this case, the maximum IL of three tapers is set as IL ≤ 0.05 dB and then the taper lengths can be optimised. It can be noted from Fig. 18(a) that the ILs of the TM_0_ ~ TM_2_ and TE_0_ ~ TE_3_ modes are decreased with the increase of the taper length and are lower than 0.05 dB after the taper length ≥ 6.35 μm. It should also be noted that a longer length is needed for a higher-order mode to achieve the desired IL of 0.05 dB. The length of the third taper is set to be *L*_t3_ = 6.35 μm and the propagation fields of the Poynting vector for the TE_3_ and TM_2_ modes are shown as two insets in Fig. 18(a). Similarly, the lengths of the second and first tapers are also studied and shown in Fig. 18(b,c), respectively. The lengths are chosen to be *L*_t2_ = 4.5 μm and *L*_t1_ = 1.5 μm for the second and first tapers, respectively. As stated above, the phase-matched widths of the access WGs of the TE_1_ ~ TE_5_ modes are different with the typical access-WG width of W_1_ = 400 nm. We can also add five tapers at the end of these five access WGs to widen the widths to W_1_ = 400 nm. Variations of the IL with the taper lengths are shown in Fig. 18(d). It can be noted that with the taper lengths of 750 nm, 1.2 μm, 50 nm, 50 nm, and 560 nm, the ILs of the TE_1_, TE_2_, TE_3_, TE_4_, and TE_5_ modes can be achieved to be ≤0.01 dB. The propagation fields of these TE modes are shown as insets in Fig. 18(d).

### Optimisation for PBS

**Figure 19:**
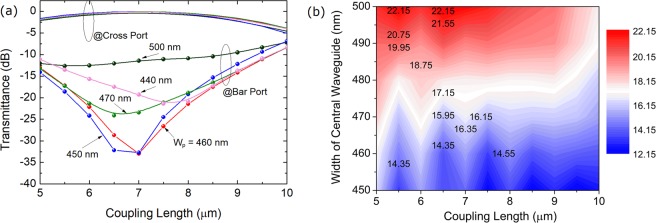
Optimisations of the PBS section. (**a**) Variations of the transmittance of the TM polarisation with the coupling length for different W_p_; (**b**) Variations of the extinction ratio for the TE polarisation with both the W_p_ and coupling length.

**Figure 20:**
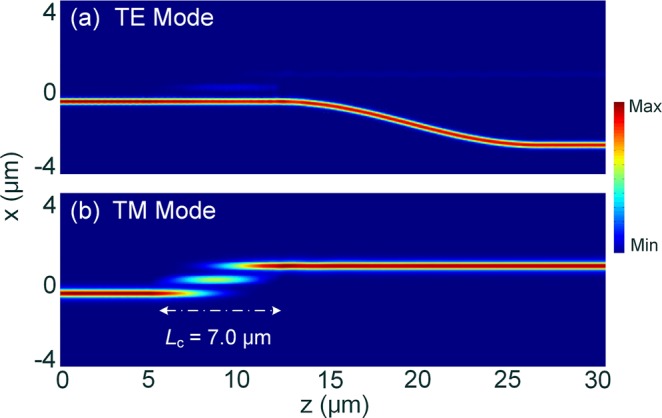
Propagation fields of the Poynting vector for PBS with (**a**) TE_0_ mode and (**b**) TM_0_ mode inputs.

The optimisation of the proposed PBS section is studied by using the 3D-FV-FDTD method. As shown in Fig. 1, the proposed PBS is based on the TWC. The widths of the two outer WGs are identical and denoted by W_1_, while that of the central WG is denoted by W_p_. In this case, the gap between the central WG and outer WGs is set to be 200 nm. For this PBS, the input TE_0_ mode from the first taper propagates along the lower WG and outputs at bar-port, while the input TM_0_ mode will be coupled to the upper WG based on the TWC and outputs at cross-port. The coupling length and the width of the central WG are optimised to achieve the desired high-performance of the PBS. Variations of the transmittance with the coupling length are shown in Fig. 19(a) for W_p_ = 440, 450, 460, 470, and 500 nm, when inputting the TM_0_ mode into the PBS. It can be noted that the lowest mode CT can be achieved around the coupling length of 7.0 μm. To further evaluate the performance of the PBS with the TE_0_ input, we calculate the ERs by varying both the coupling length and the width of the central WG, as shown in Fig. 19(b). It can be noted that the ER of the TE polarisation is increased with the increase of the W_p_ due to the increasing phase-mismatching. In this case, the coupling length and the W_p_ are chosen to be 7.0 μm and 460 nm, respectively, which can achieve the IL of 0.07 dB (0.32 dB) and ER of 32.9 dB (15.4 dB) for the TM mode (TE mode), respectively. The propagation fields of the Poynting vector are shown in Fig. 20(a,b) for the TE_0_ and TM_0_ modes, respectively. It can be observed that the input TE_0_ mode is directly propagating through the lower WG and outputs at bar-port, while the input TM_0_ mode is completely transferred from the input WG to the upper WG and outputs at cross-port. Hence, the TE_0_ and TM_0_ modes can be efficiently separated or combined by using the proposed PBS.

### Multimode Fibre-Chip Coupling and Performance Comparisons

The coupling between the high-order modes of the silicon bus-waveguide and few-mode fibre (FMF) is very challenging. Recently, several approaches have been reported to achieve the multimode fibre-chip coupling, including the vertical and edge coupling mechanisms^5,30^. For the multimode vertical-coupling, the high-order modes of an FMF can be excited by using the 2D grating couplers based mode exciter and phase shifters^31,32^. Each lobe of the high-order modes of the FMF is independently excited via 2D grating couplers based mode exciter and its phase state is tuned by using the phase shifters. Six and eight orthogonal LP modes of the FMF have been excited based on the four-grating coupler based mode exciter^31–33^. However, a high coupling-loss of ~3 dB needs to be improved and the bandwidth may be limited by these 2D gratings. For the multimode edge-coupling, the inverse tapers are of great promise to realise a low loss and broadband multimode coupler. By using two inverse tapers incorporated with a Y-junction, both the LP_01_ and LP_11a_ modes of an FMF can be coupled to a silicon chip^34^. However, this structure cannot handle any vertical high-order LP modes owing to its single-mode property along the vertical direction. Another edge-coupling approach could be the use of an inverse taper embedded in a silicon nitride (SiN) strip waveguide^35^. Six orthogonal spatial and polarisation modes (i.e. TE_11_, TE_21_, TE_31_, TE_41_, TM_11_, and TM_12_) can be coupled with the LP_01_, LP_11a_, and LP_11b_ modes of the FMF, which can achieve an ultra-low loss <0.2 dB for all these modes. A broad bandwidth has also been obtained for this structure. Hence, the efficient multi-mode coupling between the 2D silicon waveguide in this case and an FMF can be yielded by carefully optimising an inverse taper embedded in a SiN strip waveguide. In addition, our proposed hybrid (De)MUX can not only be used in optical fibre transmission systems, but also can be used in on-chip MDM systems.

The performances of the previously reported and our proposed hybrid (De)MUXs are summarised in Table 2. It can be noted that the footprint of our proposed 10-mode hybrid (De)MUX is the minimum size compared with the reported 8-, 10- and 11-mode (De)MUXs. In addition, the length of our proposed 10-mode hybrid (De)MUX is less than a quarter of the reported 10-mode hybrid (De)MUX. Although the mode CT is not less than the reported 10-mode hybrid (De)MUX, it can be dramatically reduced by implementing the TM-pass polarisers for our proposed device. Therefore, our proposed 10-mode hybrid (De)MUX is comparatively more compact than the reported ones and can be applicable to the practical telecommunication systems.

**Table 2:** Comparison of reported hybrid (De)MUXs and our proposed structure.

Reference/Year	Structure	Mode Number	Footprint	Bandwidth (nm)	Crosstalk (dB)
^22^/2013	Micro-ring resonators + ADCs	3 TE-modes	0.11 mm^2^	C-band	<−16
^23^/2013	Cascaded ADCs	4 TM-modes	~100 μm	20	<−23
^24^/2013	Grating-assisted ADCs	4 TE-modes	28 μm	<11.8@3 dB	<−22.6
^26^/2014	Cascaded-ADCs	8 hybrid modes	>300 μm	30	<−10
^27^/2014	Cascaded-ADCs	8 hybrid modes	336 μm	100	~−20
^25^/2014	Cascaded dual-core ACs	10 hybrid modes	>300 μm	90	<−15
^28^/2018	Cascaded-ADCs + SWGs	11 TE-modes	507 × 5.29 μm^2^	50	<−15.4
Our work	Cascaded ADCs via multi-phase matching	10 hybrid modes	74.75 μm	>85@1 dB; >100@3 dB	<−11.4

## Conclusion

In conclusion, we have proposed and optimised a compact 10-mode hybrid (De)MUX based on three cascaded ADCs based sections, three tapers, and a PBS for hybrid MDM and PDM systems. The bus WGs of three ADC-sections were optimised according to the phase-matching conditions for the TM_1_, TM_2_, and TM_3_ modes and then the phase-matching conditions for TE_1_ ~ TE_5_ modes were achieved by varying the widths of the access WGs based on the 3D-FV-FDTD method. The total coupling-length of three cascaded ADC-sections for the TM_1_ ~ TM_3_ and TE_1_ ~ TE_5_ modes were calculated to be 55.4 μm with the high MCE of >95%. The operation bandwidths have also been studied for three cascaded ADC-sections and the results show that the optimised ADC-sections can operate over the C-band with ultra-low ILs and high ERs. The tapers between the adjacent ADC-sections and PBS were optimised and the taper lengths were achieved to be 1.5, 4.5, and 6.35 μm for the first, second, and third tapers with the IL of ≤0.05 dB. Compared with the conventional cascaded-ADCs based hybrid (De)MUXs, the number of the tapers can be significantly decreased, which can further reduce the total IL of the proposed device. The PBS used for (de)multiplexing the TM_0_ and TE_0_ modes have been optimised with a compact length of 7.0 μm and the IL of 0.07 dB (0.32 dB) and ER of 32.9 dB (15.4 dB) for the TM mode (TE mode), respectively. The proposed schematic of the hybrid (De)MUX can be extended for more number of the operation modes, which offers the application in the hybrid MDM-PDM networks.

## Methods

The effective indices of the silicon waveguides are calculated by using the full-vectorial finite element method (FV-FEM). The refractive indices of the silicon and silicon oxide are set to be 3.47548 and 1.46, respectively at the wavelength of 1550 nm. The phase-matching conditions of the isolated access and bus waveguides are calculated by using the FV-FEM, while those of the composite asymmetric directional couplers (ADCs) are calculated by using the 3D full-vectorial finite difference time domain (3D-FV-FDTD) method. The mode conversion efficiency (MCE) and coupling lengths of three cascaded-ADCs based sections are optimised based on the 3D-FV-FDTD method. The propagation fields of the Poynting vector for ten modes are calculated by using the 3D-FV-FDTD method. The bandwidths of three cascaded ADC-sections are calculated by using the 3D-FV-FDTD method. The optimisations of the tapers and PBS are achieved by using the 3D-FV-FDTD method.
